# In Vitro and In Vivo Characterization of Selected Fluorine-18 Labeled Radioligands for PET Imaging of the Dopamine D3 Receptor

**DOI:** 10.3390/molecules21091144

**Published:** 2016-08-29

**Authors:** Natascha Nebel, Simone Maschauer, Torsten Kuwert, Carsten Hocke, Olaf Prante

**Affiliations:** Molecular Imaging and Radiochemistry, Department of Nuclear Medicine, Friedrich Alexander University (FAU), Erlangen 91054, Germany; natascha.nebel@uk-erlangen.de (N.N.); simone.maschauer@uk-erlangen.de (S.M.); torsten.kuwert@uk-erlangen.de (T.K.); carsten.hocke@uk-erlangen.de (C.H.)

**Keywords:** positron-emission-tomography (PET), fluorine-18 (F-18; ^18^F), dopamine-D3 receptor (D3), autoradiography

## Abstract

Cerebral dopamine D3 receptors seem to play a key role in the control of drug-seeking behavior. The imaging of their regional density with positron emission tomography (PET) could thus help in the exploration of the molecular basis of drug addiction. A fluorine-18 labeled D3 subtype selective radioligand would be beneficial for this purpose; however, as yet, there is no such tracer available. The three candidates **[^18^F]1**, **[^18^F]2a** and **[^18^F]2b** were chosen for in vitro and in vivo characterization as radioligands suitable for selective PET imaging of the D3 receptor. Their evaluation included the analysis of radiometabolites and the assessment of non-specific binding by in vitro rat brain autoradiography. While **[^18^F]1** and **[^18^F]2a** revealed high non-specific uptake in in vitro rat brain autoradiography, the D3 receptor density was successfully determined on rat brain sections (*n* = 4) with the candidate **[^18^F]2b** offering a B_max_ of 20.38 ± 2.67 pmol/g for the islands of Calleja, 19.54 ± 1.85 pmol/g for the nucleus accumbens and 16.58 ± 1.63 pmol/g for the caudate putamen. In PET imaging studies, the carboxamide **1** revealed low signal/background ratios in the rat brain and relatively low uptake in the pituitary gland, while the azocarboxamides **[^18^F]2a** and **[^18^F]2b** showed binding that was blockable by the D3 receptor ligand BP897 in the ventricular system and the pituitary gland in PET imaging studies in living rats.

## 1. Introduction

Positron emission tomography (PET) is a molecular imaging modality, allowing, in particular, the non-invasive assessment of cerebral neurotransmission [1,2]. PET depends on the availability of radiopharmaceuticals carrying a positron emitter. Fluorine-18 is a positron emitting nuclide offering properties favourable for PET imaging. Among these is its comparatively long half-life of 110 min, offering sufficient time for its transportation from the site of its production to the site of its application as well as for radiosynthesis and image acquisition with a reasonably limited patient exposure to radiation [2,3,4].

A rational starting point for radioligand design is choosing structural features from known and well characterized ligands [5,6]. Low dense receptors in the central nervous system demand binding affinities in the sub-nanomolar range to distinguish specific from non-specific binding [7]. Non-specific binding needs to be reduced by good target selectivity and a high specific activity of the radioligand candidate. Low specific activities can cause saturation of binding sites and may even lead to pharmacological or toxic side effects [2]. Rapid metabolism of radioligands can lead to problems during image acquisition, especially when the metabolites contain the label and penetrate the blood-brain-barrier [8,9]. Other important prerequisites for radioligands suitable for in vivo use are fast blood clearance and low non-specific binding to proteins in plasma [5,7].

The blood-brain-barrier (BBB) is a restrictive barrier of endothelial cells connected through tight junctions, allowing passive diffusion mainly to small lipid-soluble molecules [10,11]. Compounds excluded from passive diffusion may cross the barrier through active transport by highly selective transporters or vesicular mechanisms [10,12]. The strategy of increasing a compound’s lipophilicity to make it more brain penetrant enhances non-specific binding and can also increase its chance of becoming a substrate for efflux transporters [13,14]. Waste and toxins are actively carried back into the capillary blood system by transporters mostly localized at the luminal membrane of the BBB endothelial cells [10]. One of these is the para-glycoprotein (P-gp), also known as multi drug resistance protein (MDR1) [14,15].

The dopamine D3 receptor (D3) belongs to the family of the dopamine D2-like receptors. Its stimulation generally decreases cell excitability [16,17]. Addressing dopamine D2-like receptors is the mode of action of many psychostimulants [18,19]. The D3 receptor in particular plays a role in drug seeking and appears to be up-regulated in the abuse of such [20,21,22]. Predominantly found in limbic areas such as the nucleus accumbens and the islands of Calleja, the D3 receptor is, therefore, a prime target for research on drug addiction [23,24,25].

**Figure 1:**
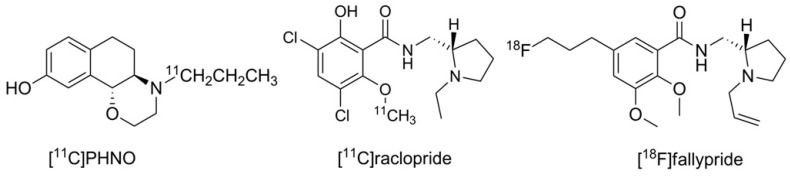
Structures of established dopamine D3 imaging agents for PET.

Until now, the D3 preferring carbon-11 labeled naphtoxazine [^11^C]PHNO (t_1/2_ = 20 min.) is the only radiopharmaceutical in use for selectively imaging the D3 receptor [26,27]. Furthermore, the D2/D3 non-selective radiotracers [^11^C]raclopride and [^18^F]fallypride may also be used for this purpose when non-radioactive D2-ligands with high affinity are pre-administered to diminish the signal of D2 binding in the PET recordings [28,29,30]. The structures of the substances in current use for PET D3 imaging are shown in Figure 1.

The development of a truly D3 selective imaging agent would reduce uncertainties in the recorded images arising from potential residual D2 binding. Furthermore, a fluorine-18 labeled ligand would be beneficial regarding its well-balanced half-life as described above. However, various efforts in this direction have not yet offered any suitable D3 radioligand [27,31,32].

**Figure 2:**
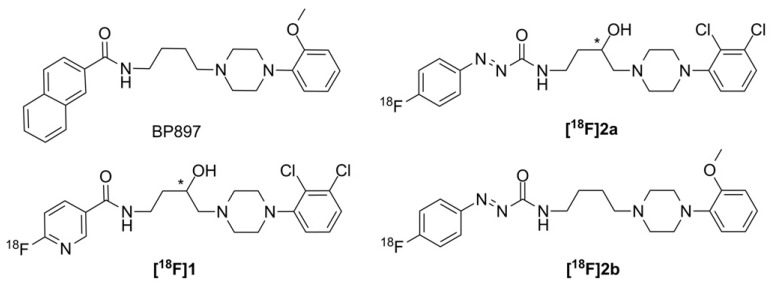
Structure of BP897 and of 4-phenylpiperazines turned into fluorine-18 labeled ligands.

Some of the ongoing attempts to develop a fluorine-18 labeled D3 subtype selective PET ligand focus on modifications of 4-phenylpiperazines, a structural family of potent D3 selective ligands including the well-known D3 partial agonist BP897 [20,31,33,34,35]. In our previous work, we focused on the development of D3 selective radioligands derived from the lead BP897, resulting in a series of fluorinated pyridinylcarboxamides and their phenylazo analogues [36,37,38]. From this series of compounds, we chose the most promising D3 radioligand candidates **[^18^F]1**, **[^18^F]2a**, and **[^18^F]2b**, as illustrated in Figure 2 together with the lead BP897, to be included in the present study.

All three ligands showed a single-digit binding affinity for the D3 receptor (K_i_(D3) = 1.9 nM (**[^18^F]1** [37]), 3.6 nM (**[^18^F]2a** [38]) and 3.5 nM (**[^18^F]2b** [36]) and a ≥ 10 fold preference over the D2 subtype. Characteristic of the radiosyntheses of all three ligands is their synthesis via [^18^F]fluorination by nucleophilic aromatic substitution, starting from precursors bearing electron withdrawing groups such as the shared carboxamide, either the pyridinyl or azo function and suitable leaving groups. The radiosynthesis of **[^18^F]1** has not been published previously, whereas the radiosyntheses of **[^18^F]2a** and **[^18^F]2b** have already been studied in detail and were successfully optimized [38].

The aim of this study was the in vitro and in vivo characterization of **[^18^F]1**, **[^18^F]2a** and **[^18^F]2b** as potential PET tracers and their use in quantification of the dopamine D3 receptor subtype. The main result of this study offers **[^18^F]2b** as the protruding candidate with which the in vitro quantification of D3 receptor expression succeeded. All three candidates were studied with respect on lipophilicity, stability in serum, and protein binding in vitro, and dopamine D3 subtype selective binding in vivo in rat brain areas, with a special focus on areas unprotected from the blood-brain-barrier, such as the pituitary gland and brain ventricles, using ex vivo autoradiography and small animal PET imaging.

## 2. Results

### 2.1. Radiosyntheses

The radiosynthesis of **[^18^F]1** was adapted from previously reported fluorine-18 labeled pyridinyl carboxamides [39]. **[^18^F]1** was obtained in a radiochemical yield of 80% (determined from a sample withdawn from the reaction mixture) and was isolated radiochemically pure in an overall non-decay corrected radiochemical yield of 15% ± 3% in a total synthesis time of 60–70 min and specific activities of 33 ± 14 GBq/µmol (*n* = 6). The radiosyntheses of the candidates **[^18^F]2a** and **[^18^F]2b** from the phenylazo type were performed as previously published [38]. The purified compounds were isolated in non-decay corrected radiochemical yields of 12% ± 2% in 40–50 min. The specific activity of **[^18^F]2a** was 57 ± 34 GBq/µmol (*n* = 6) and **[^18^F]2b** reached 167 ± 62 GBq/µmol (*n* = 6). Starting from [^18^F]fluoride (0.6–1 GBq), the radiosyntheses of all three candidates guaranteed sufficient radiochemical yield and high specific activity for the following in vitro and in vivo experiments.

### 2.2. Determination of LogD_7.4_, Stability and Binding to Proteins

The pyridinylcarboxamide **[^18^F]1** had a log D_7.4_ of 2.50 ± 0.04 (*n* = 6). The log D_7.4_ coefficients of the aza bearing compounds have been previously reported with a value of 2.56 ± 0.01 (*n* = 6) for the 2,3-dichloro substituted **[^18^F]2a** and a log D_7.4_ of 2.10 ± 0.10 (*n* = 6) for the methoxy bearing **[^18^F]2b** [36,38]. Calculated log D coefficients (log D_7.4calc._) were compared to the experimentally determined values in Table 1, as there is often a discrepancy between both methods [40,41].

At a physiological pH the protonation status of the piperazine nitrogen facing the alkyl chain, is calculated to be abundant in the protonated species (77%) for **[^18^F]2b** and deprotonated for **[^18^F]1** and **[^18^F]2a** (Table 1).

**Figure 3:**
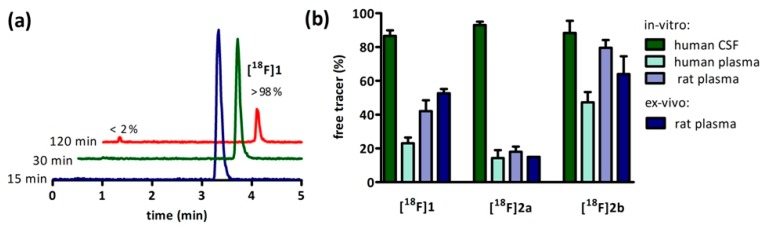
In vitro stability and free fraction of **[^18^F]1**, **[^18^F]2a** and **[^18^F]2b** in CSF, human and rat plasma. (**a**) Representative radiochromatogram of **[^18^F]1** demonstrating high in vitro stability in human serum at 37 °C; (**b**) Free fraction of the radioligand in human CSF and rat plasma after 30 min of incubation or i.v. injection of the respective radioligand. In Vitro binding experiments were performed in triplicates and the data on the free fraction in rat plasma after i.v. injection were the result of two separate determinations performed in triplicates. The values are expressed as mean ± SD.

The in vitro stability of **[^18^F]2a** and **[^18^F]2b** in human serum have been previously reported, whereby no decomposition was observed over 120 min [36,38]. Candidate **[^18^F]1** showed a decomposition of ≤2% after 120 min (Figure 3a). Thus, all three ligands indicated excellent stability in human serum in vitro, predicting high stability in blood in vivo.

The binding to proteins in plasma and cerebrospinal fluid (CSF) in vitro and determined in rat plasma after i.v. injection of the tracer is shown in Figure 3b. Interestingly, the radioligands were bound only to 5%–15% to proteins in human CSF. The binding of the candidates **[^18^F]1** and **[^18^F]2b** to human plasma was 20%–30% higher than in rat plasma. The methoxy substituted candidate **[^18^F]2b** offered 80% free radioligand in studies with rat plasma. The percentage of free radioligand for the 2,3-dichloro substituted **[^18^F]1** is more moderate with 42% and **[^18^F]2a**, additionally bearing the aza moiety, indicated only 18% free radioligand. Taken together, **[^18^F]2b** revealed the highest free fraction in plasma, which should be translated into higher brain uptake of **[^18^F]2b** in vivo when compared to the analogs **[^18^F]1** and **[^18^F]2a**.

**Figure 4:**
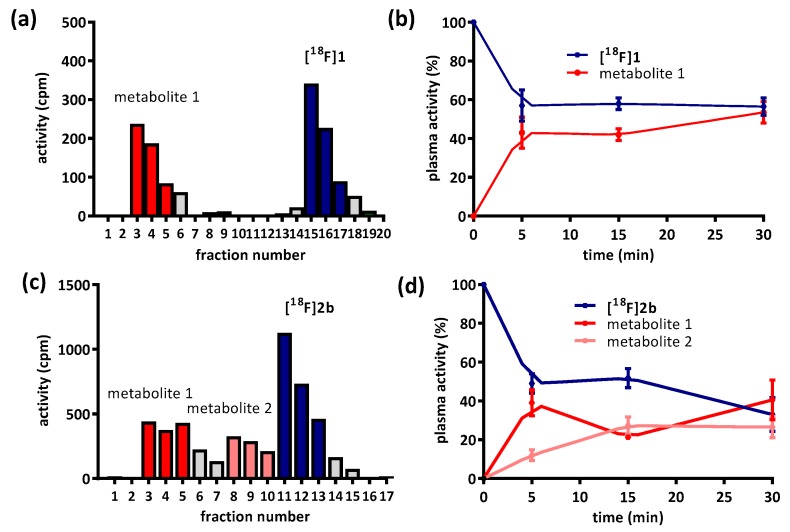
Stability of radioligands **[^18^F]1** and **[^18^F]2b** in blood of living rats under isoflurane anesthesia. (**a**,**c**) Radio-chromatogram obtained by HPLC of rat plasma extract taken 15 min after i.v. administration of the radioligand; (**b**,**d**) Fraction of intact tracer and radiometabolite as a function of time. Points represent the mean ± SEM of duplicates.

Concerning the in vivo stability, the carboxamide **[^18^F]1** was compared with the azocarboxamide **[^18^F]2b** (Figure 4). The in vivo stability of **[^18^F]2a** could not be determined due to the low count rate of the plasma samples. **[^18^F]1** comprised 57% intact tracer and a single unknown hydrophilic radiometabolite that occured at 15 min post intravenous injection as illustrated in Figure 4a. The percentage of intact **[^18^F]1** (~60%) and radiometabolite (~40%) remained nearly constant over the total sampling time (Figure 4b). The samples of azocarboxamide **[^18^F]2b** also featured a hydrophilic radiometabolite in the fractions 3, 4 and 5 (Figure 4c). However, in case of the azocarboxamide the formation of a second less hydrophilic radiometabolite, which was eluted before the intact tracer, occured. Further the metabolisation of **[^18^F]2b** continued until the end of the sampling with 49% intact tracer after 5 min and 37% intact tracer after 30 min (Figure 4d). Obviously, the carboxamide **[^18^F]1** revealed a slightly higher stability in vivo than the azocarboxamide **[^18^F]2b**, however, this observation could be due to different reactivities at the phenylpiperazine part of the molecules.

### 2.3. In Vitro Autoradiography

**Figure 5:**
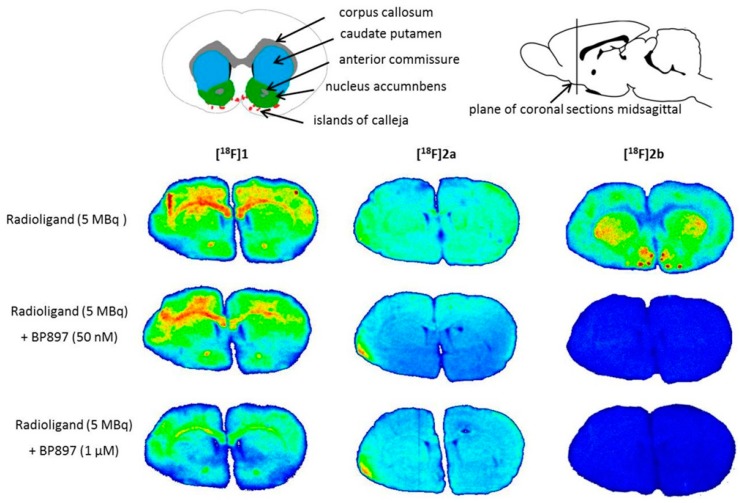
In vitro autoradiography of coronal rat brain sections after incubation with the respective radioligand and in presence of the D3 receptor ligand BP897. Regions visible in the autoradiograms were drawn onto the coronal illustration provided by the rat brain atlas (Paxinos and Watson [42]).

The rat brain sections incubated with compound **[^18^F]1** exhibited pronounced non-specific binding in the corpus callosum and anterior commissure as well as displacable binding by BP897 in the caudate putamen (Figure 5). Compound **[^18^F]2a** showed homogenous non-specific binding throughout the whole rat brain sections. In contrast to these observations, D3 specific binding was visible with **[^18^F]2b** in the caudate putamen, nucleus accumbens and Islands of Calleja, substantiated through complete displacement of the binding through BP897 (Figure 5). The experiment with **[^18^F]2b** has been previously published [36] and was reproduced for the present study.

**Figure 6:**
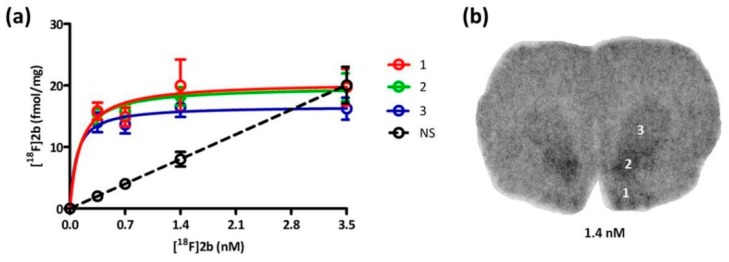
Saturation binding experiment with **[^18^F]2b** using rat brain slices. (**a**) Specific binding of **[^18^F]2b** at the islands of calleja (1), nucleus accumbens (2) and caudate putamen (3) determined by a saturation binding assay. Non-specific binding (NS) was determined through co-incubation with BP897. Points represent the mean ± SD of four independent experiments performed in duplicates; (**b**) Representative autoradiogram of a coronal rat brain section incubated with 1.4 nM of **[^18^F]2b**.

The specific binding of **[^18^F]2b** in rat brain sections was determined by saturation binding assays as illustrated in Figure 6a. The results on the maximal receptor density (B_max_) are listed in Table 2.

**[^18^F]2b** exhibited a maximal receptor density (B_max_) of 20.38 pmol/g for the Islands of Calleja and 19.54 pmol/g for the nucleus accumbens. The caudate putamen offered a B_max_ of 16.58 pmol/g. **[^18^F]2b** had a K_d_ value near 0.1 nM for all three regions.

### 2.4. Dynamic PET Recordings

**Figure 7:**
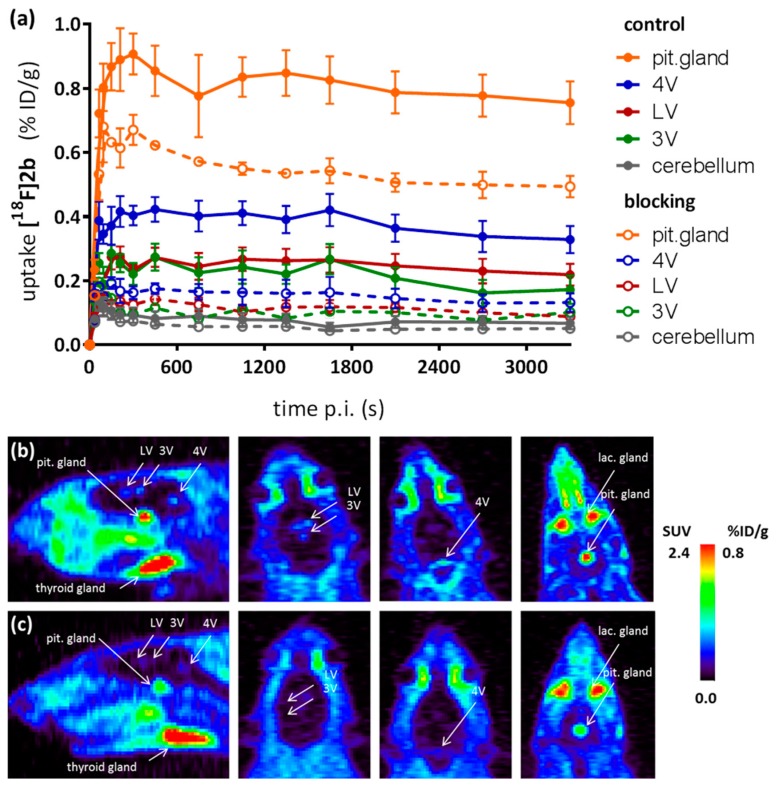
PET imaging study in rats using **[^18^F]2b**. (**a**) TACs post i.v. injection of **[^18^F]2b** in rats. Points represent the mean ± SEM control animals (*n* = 4) and animals with co-injection of BP897 (*n* = 2); (**b**) Exemplary recording showing the frame 35–45 post radioligand injection and in presence of BP897 (**c**). Regions were defined with help of the rat brain atlas [42].

Dynamic PET recordings of rats injected with the most promising radioligand **[^18^F]2b** were performed. Figure 7a shows the dynamic time-activity curves which displayed a low uptake in the cerebral matter for **[^18^F]2b**, reaching a maximum at 65 s post intravenous injection of the radioligand at 0.14 %ID/g and wash-out to 0.07 %ID/g over the 60 min of recording.

The uptake and wash-out in the cerebral matter were not significantly influenced by blocking studies with BP897. A higher uptake was registered in the pituitary gland (pit. gland), attaining an uptake of 0.94 %ID/g after approximately 5 min followed by slow wash-out to 0.8 %ID/g at 30 min p.i., and reaching 0.76 %ID/g at end of the recording. Blocking with BP897 significantly decreased initial and mean uptake by 35% with no influence in wash-out kinetics. This was also observed in distinct patches of the ventricular system, belonging to the third (3V), fourth (4V) and lateral ventricle (LV), nicely visible in the recordings taken 35 min postinjection of **[^18^F]2b** (Figure 7b). The highest ventricular uptake was registered in the fourth ventricle with an initial uptake of 0.4 %ID/g that was constant over the 60 min of recording and could be blocked by 50% with BP897. The third and lateral ventricle attained an uptake of approximately 0.25 %ID/g.

The registration of in vivo uptake in the ventricluar system during PET recordings is an interesting finding with this compound class, so although compounds **[^18^F]1** and **[^18^F]2a** were not promising in vitro due to their high nonspecific uptake seen by in vitro rat brain autoradiography, dynamic PET recordings were acquired with all three tracers to study if the in vitro results are mimicked by the in vivo results.

**Figure 8:**
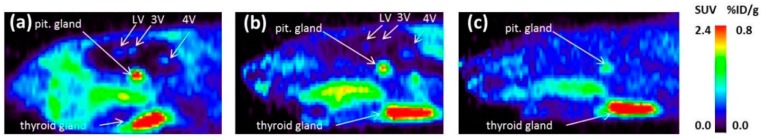
Exemplary midsagittal view of PET recordings at ~30 min post radioligand injection. (**a**) **[^18^F]2b** (**b**) **[^18^F]2a** and (**c**) **[^18^F]1**.

**Figure A1:**
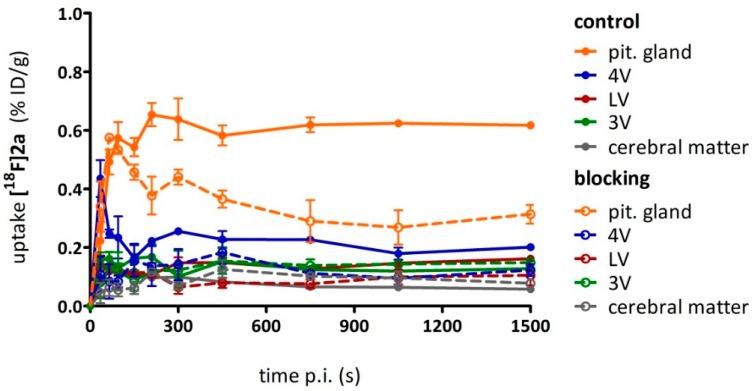
ACs post i.v. injection of **[^18^F]2a** in rats, as obtained by small animal PET analysis. The data points represent the mean ± SEM of two independent experiments. The blocking study was performed by co-administration of BP897 (1 mg/kg, *n* = 2).

Figure 8 offers a direct visual comparison of the in vivo behaviour of the three candidate radioligands. Compound **[^18^F]2a** showed similar tracer distribution in the rat brain in vivo as **[^18^F]2b** (Figure A1, Figure 8a,b), with specific ventricular uptake and uptake in the pituitary gland (0.6 %ID/g at 25 min p.i.) that was only slightly lower than that of **[^18^F]2b** (Figure 8). The high thyroidal uptake of all three tracers, as visualized in Figure 8, was non-specific, as it was not significantly reduced by coinjection of BP897 (data not shown).

**Figure A2:**
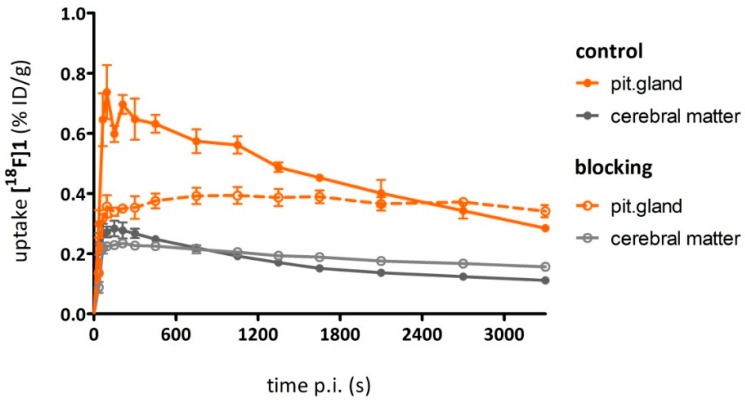
TACs post i.v. injection of **[^18^F]1** in rats, as obtained by small animal PET analysis. Points represent the mean ± SEM of two independent experiments. The blocking study was performed by co-administration of BP897 (1 mg/kg, *n* = 2).

The TACs of **[^18^F]1** also showed uptake in the pituitary gland (0.45 %ID/g at 30 min p.i.) but to a much lower extent than the uptake of **[^18^F]2a** and **[^18^F]2b**. However, the uptake of **[^18^F]1** in the pituitary gland was most probably non-specific because there was no difference in uptake compared to the blocking conditions with BP897 at late points after tracer injection (Figure A2). Moreover, **[^18^F]1** showed homogenous distribution of radioactivity in the rat brain and increased background when compared to **[^18^F]2a** and **[^18^F]2b**, such that a signal in the ventricles was undetectable due to high background values (Figure A2 and Figure 8c). Uptake of **[^18^F]1** in the cerebral matter reached a maximum at 0.25 %ID/g after 50 seconds and decreased to 0.17 %ID/g over the course of the recordings, but was still almost twofold higher than that of **[^18^F]2a** and **[^18^F]2b**. Noteworthy, the uptake in cerebral matter in case of the azo analogues **[^18^F]2a** and **[^18^F]2b** was very low, constant over time after injection and comparable to uptake in the cerebellum.

### 2.5. Ex-Vivo Autoradiography Study

**Figure 9:**
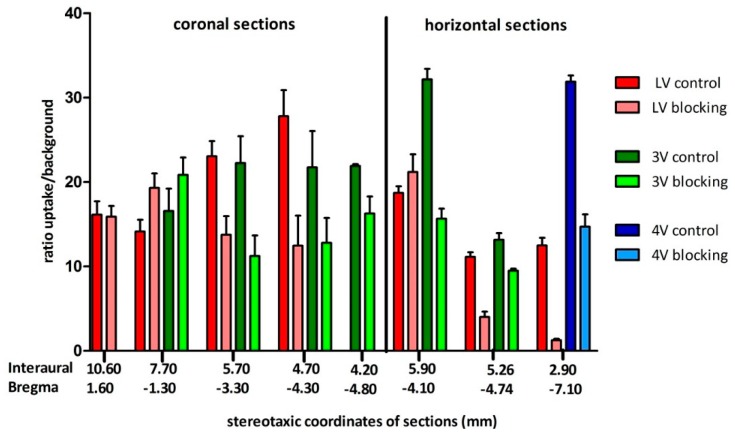
Ratio ROI/background of coronal and horizontal ex vivo rat brain sections from Figure A3. The value of each section represents the mean ± SD of two animals (coronal: *n* = 2; horizontal: *n* = 2) with triplicates of each plane and mean of mirroring ventricles. The sections were assigned to Figures in the rat brain atlas with stereotaxic coordinates (Paxinos and Watson [42]).

Ex-vivo sections with the coordinates (Interaural 10.60, Bregma 1.60) and (Interaural 7.70, Bregma −1.30) in the rat brain atlas [42] exhibited a ratio near 15 for ROI/background and no displacement of **[^18^F]2b** by BP897 in the lateral (LV) and third ventricle (3V) (Figure 9).

The section with the coordinates (Interaural 5.70, Bregma −3.30) exhibited a ratio of 22 (ROI/background) for both lateral and third ventricle and a displacement of **[^18^F]2b** by 40%–50%. Highest ventricular uptake of **[^18^F]2b** in the coronal sections was with (Interaural 4.70, Bregma −4.30) reaching a ratio of nearly 30 and displacement of 60% with BP897 for the lateral ventricle. The ratio of the third ventricle remained nearly constant throughout the coronal sections. Highest uptake of the third ventricle was with horizontal section (Interaural 5.90, Bregma −4.10) with a ratio of 32 ROI/background, displacable by 50% through BP897. The fourth ventricle of section (Interaural 2.90, Bregma −7.90) exhibited similar results. These data revealed D3 receptor-mediated binding of **[^18^F]2b** in the ventricles that was only partly displacable by BP897.

### 2.6. In Vitro Assessment of P-gp Mediated Efflux

**Figure 10:**
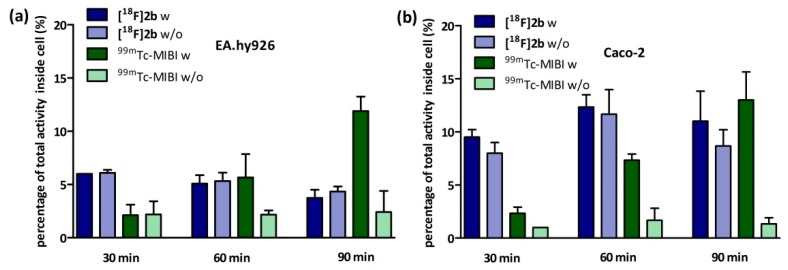
Determination of P-gp substrate specificity by studying cellular uptake of **[^18^F]2b** in comparison with ^99m^Tc-MIBI. (**a**) Influence of cyclosporin-A on cellular concentration of ^99m^Tc-MIBI and **[^18^F]2b** in EA.hy926 cells and (**b**) in Caco-2 cells (w: in the presence of cyclosporin-A (5 nM), w/o: without cyclosporin-A).

Due to the lack of uptake of **[^18^F]2b** in D3-rich brain areas, such as the striatum, nucleus accumbens or the Islands of Calleja, we studied the substrate specificity of **[^18^F]2b** for P-gp. In EA.hy926 and Caco-2 cells the known P-gp substrate ^99m^Tc-MIBI is accumulated in presence of the P-gp inhibitor cyclosporin-A. As demonstrated in Figure 10, the presence of cyclosporin-A did not increase the uptake of **[^18^F]2b** in both cell lines. The percentage of **[^18^F]2b** inside the cells was twice as high in Caco-2 cells (10%) as with EA.hy926 (5%).

**Figure 11:**
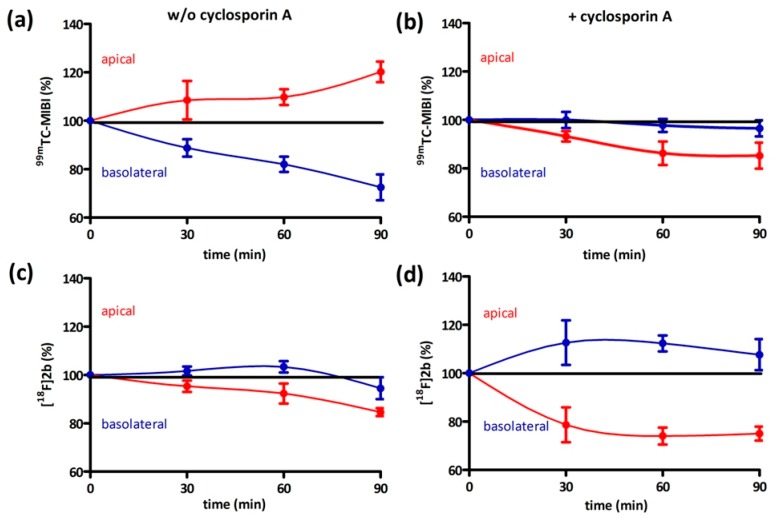
Bi-directional transport assay. (**a,c**) Bi-directional transport of ^99m^Tc-MIBI and **[^18^F]2b** in absence of cyclosporine-A. (**b,d**) Bi-directional transport in presence of cyclosporine-A. Points represent mean ± SEM of four independent experiments.

In addition, a bi-directional transport assay was performed starting with equal concentration in an apical and basolateral compartment (Figure 11). ^99m^Tc-MIBI steadily increased in the apical compartment to 120% of the initial concentration after 90 min in abscence of cyclosporin. The concentration in the basolateral compartment was decreased to 70% (Figure 11a). In presence of cyclosporin-A the concentration of the basolateral compartment remained nearly constant and 90% of the initial concentration could be found in the apical compartment after 60 and 90 min (Figure 11b). **[^18^F]2b** exhibited similar behaviour in absence of cyclosporin-A as ^99m^Tc-MIBI in presence of cyclosporin (Figure 11c). The concentration in the basolatreal compartment stayed nearly constant at 100% of the inital value and at 90% in the apical compartment. In presence of cyclosporin-A the basolateral concentration also stayed nearly constant, but the concentration of the apical compartment dropped to 80% after 30 min (Figure 11d), showing a slightly increased accumulation of **[^18^F]2b** in the cells, possibly due to facilitated diffusion of the tracer into the cells caused by cell membrane damages in presence of cyclosporin-A.

## 3. Discussion

There are commonly accepted criteria for radiochemical purity of >95% and acceptable radiochemical yields in the range of 20%–40% for ^18^F-labeled tracers. The radiosyntheses of the three candidates **[^18^F]1**, **[^18^F]2a** and **[^18^F]2b** for PET imaging of the dopamine D3 receptor in the present study fulfilled these criteria adequately. Concerning the obtained overall radiochemical yields of about 10%–15%, the losses during radiosynthesis accounted to azeotropic drying of the [K^+^ ⊂ K_222_][^18^F]F^−^ complex, adhesion to reaction vessels and transfer utensils in total. Major loss occurs during the final separation on the HPLC column, only one fourth of the injected activity was recovered and could not be increased with alternative semi preparative columns (RP C8, RP C18). However, starting with [^18^F]fluoride of 0.6–1 GBq the overall non-decay-corrected yield for all three candidates was sufficient for preclinical evaluation.

Besides radiochemical purity, the specific activity of a radiotracer is of great importance to preclude the saturation of binding sites. Published values for the specific activity of ^18^F-labeled PET imaging probes using carrier free ^18^F-fluoride are generally in the range of 50–500 GBq/µmol [2,43]. Fluorine-18 labeled radioligands developed for the central nervous system exhibiting specific activities ranging from 11–135 GBq/µmol were successful in addressing the dopamine transporter and the monoamine-oxidase-A in PET studies with rats [44,45]. The established radiotracer [^11^C]PHNO for human PET imaging of the less abundantly expressed dopamine D3 receptor has been applied in studies with specific activities of 31–67 GBq/µmol [26,46]. Furthermore, a PET study in mice with D2/D3 non-selective [^18^F]fallypride found no decline in specific binding associated with mass effects with specific activities between 50–140 GBq/µmol [47]. Whereas the average specific activity of 33 GBq/µmol for **[^18^F]1** and 57 GBq/µmol by **[^18^F]2a** were below or in the lower range of the above-given ideal values, the specific activity of **[^18^F]2b** was with 167 GBq/µmol well acceptable. Disturbed binding due to limitations in specific activity of **[^18^F]1**, **[^18^F]2a** and **[^18^F]2b** in the present study can therefore be considered highly unlikely.

The experimentally determined Log D_7.4_ values of **[^18^F]1**, **[^18^F]2a** and **[^18^F]2b** were between 2.10 and 2.56, while the calculated values were with 2.63–3.89 higher (Table 1). The 2-methoxyphenylpiperazinyl substituted azacarboxamide **[^18^F]2b** has the lowest Log D_7.4_ value of 2.10 of the three compounds strudied herein. The two 2,3 dichloro-substituted candidates share very close values, whereas the pyridinylcarboxamide **[^18^F]1** showed a value of 2.50 followed by the azocarboxamide **[^18^F]2a** with 2.56. The calculated pKa of Table 1 seems solemnly dependent on the substitution of the phenylpiperazine, with 6.95 for **[^18^F]1** and **[^18^F]2a** (2,3-chloro-substituted) for the piperazine nitrogen facing the alkyl chain. **[^18^F]2b** with 7.93 would be predominantly protonated at physiological pH of 7.4, which is in accordance with the experimental value, deeming it the most hydrophilic of the compounds. **[^18^F]1** and **[^18^F]2b** show opposite protonation species but their calculated as well as experimentally determined log D_7.4_ values were in the range of 2–3.5, which had been described as an optimal range for crossing of the blood-brain-barrier [2,48].

Non-specific binding to tissue and proteins often accompanies the window of lipophilicity needed to penetrate the brain and an increase of this variable with higher LogD values was observed in vitro and ex vivo. **[^18^F]2b** offers the highest percentage of available radioligand with 80% of the administered dose found unbound to proteins in plasma (Figure 3b). This proposedly has a positive influence on the target/plasma ratio needed to obtain a good and specific signal and increases the availability of free tracer potentially crossing the blood-brain-barrer [2,13]. The compounds **[^18^F]1** and **[^18^F]2a** both exhibit a higher lipohilictiy and are, thus, far less available for transport into the brain with 58% of the injected dose bound to proteins in plasma for **[^18^F]1** and 82% of **[^18^F]2a**.

Besides non-specific binding of the radioligands, their availability is also limited by metabolism. The hydrophilic metabolite of **[^18^F]1** and **[^18^F]2b** (Figure 4a,c) was previously reported to correspond to pyridinylcarboxamides and was predicted not to cross the blood-brain-barrier [35]. Nevertheless, images from the PET recordings were interpreted with regard to signal potentially emanating from radiometabolites; especially the radiometabolite detected in fractions 8–10 of the sampling from **[^18^F]2b** could be critical, since it was eluted directly before the intact tracer itself (Figure 4c). The identity of the radiometabolite was not determined, but likely derived from *O*-demethylation of the phenylpiperazines methoxy function, a common reaction catalyzed by cytochrome P450 [9,49,50,51].

Similar non-specific binding of **[^18^F]1** and **[^18^F]2a** to proteins was observed in the in vitro autoradiography of rat brain sections. The sections incubated with **[^18^F]1** showed elevated binding at the corpus callosum and anterior commissure, which are white-brain matter structures consisting mostly of glial cells and axons coated with a myelin sheath. The uptake of **[^18^F]1** in the region of the caudate nucleus, the putamen, and the nucleus accumbens is probably unspecific, since 50 nM BP897 did not show any reduced binding, and 1 µM of BP897 reduced the uptake in any region throughout the brain. The even more lipophilic **[^18^F]2a** exhibits homogenous non-specific binding throughout the whole section, even in the autoradiography in the presence of BP897. In comparison, the most hydrophilic ligand **[^18^F]2b** showed clear displacement of specific binding in the dopamine-D3 receptor-rich Islands of Calleja, the nucleus accumbens, and the striatum and clearly reduced non-specific binding to tissue compared with the other candidates in the in vitro autoradiography (Figure 5). To identify the azo moiety as beneficial compared to the pyridinyl feature in in vitro autoradiography studies, a ligand differing only in these features needs to be applied. A series covering such a structure has been reported, but showed lower binding affinity towards the dopamine D3 receptor [37], impeding a direct comparison.

Based on the in vitro autoradiography studies the receptor density (B_max_) and binding dissociation constant (K_d_) of **[^18^F]2b** to the D3 receptors on brain sections of Wistar rats (350–400 g) were determined (Figure 6). The highest receptor expression was found in the Islands of Calleja with 20.38 pmol/g and nucleus accumbens with 19.54 pmol/g. The value in the striatum was slightly lower with 16.63 pmol/g (Table 2). In the literature B_max_ values of male Wistar rats (180–200 g) were determined with [^3^H]7-OH-DPAT to be 10 pmol/g in the nucleus accumbens and 5.5 pmol/g in the striatum (caudate nucleus/putamen) [29]. Another study determined the B_max_ to be 26 pmol/g in the ventral striatum (nucleus accumbens) with [^3^H]PD 128907 and 36 pmol/g with [^3^H]7-OH-DPAT of male Sprague Dawley rats weighing 200–300 g [52]. The subnanomolar K_d_ value of 0.1 nM was exhibited in all three regions evaluated, indicating that **[^18^F]2b** is a high affinity ligand for the D3 receptor, as to be expected from its previously determined K_i_ value of 3.5 nM [36].

As a complement to in vitro autoradiography, PET studies were performed to further evaluate **[^18^F]2b** as a tracer suited to selectively image dopamine D3 receptors in vivo. Interestingly, high uptake of **[^18^F]2b** was found in the pituitary gland and in distinct patches of the ventricular system, belonging to the third, fourth and lateral ventricle (Figure 7). Highest ventricular uptake was registered in the fourth ventricle with an initial uptake of 0.4 %ID/g that remained constant over the 60 min of recording and could be blocked to 50% by BP897. These findings partly confirmed previously reported in vivo PET recordings of structurally related carboxamides. Displaceable binding in the pituitary gland has been reported for the pyridinylcarboxamides. These compounds showed a cerebral uptake of 1 %ID/g in the striatum and cerebellum followed by a wash-out to 0.3 %ID/g after 30 min [35]. An interesting finding with these compounds is their in vivo uptake into the ventricular system. Although **[^18^F]1** and **[^18^F]2a** exhibited high non-specific binding in vitro, dynamic PET studies of their cerebral uptake were also performed, disclosing lowest uptake of **[^18^F]1** in the pituitary gland, but very similar in vivo results of **[^18^F]2a** as compared to **[^18^F]2b** concerning regional uptake and displacement of the binding by BP897. Brain uptake was by a factor of 1.5 smaller for **[^18^F]2a** than for **[^18^F]2b**. This is explainable by the above already discussed reduced amount of free **[^18^F]2a** in circulating plasma, due to binding to serum proteins. All three candidates failed to image the typically D3 rich Islands of Calleja and the nucleus accumbens in vivo. The uptake in cerebral matter in case of the azo analogues **[^18^F]2a** and **[^18^F]2b** was very low, comparable to uptake in the cerebellum. The binding in the pituitary gland of **[^18^F]2a** and **[^18^F]2b** and ventricles was very distinct and not perturbed by non-specific binding.

The expression of D3 receptors has been reported in areas outside the blood-brain-barrier protected cerebral matter, such as in the pituitary gland and in the ependymal cell layer of the lateral ventricle. The ependyma of the choroid plexus is strongly immunoreactive and showed specific D3 receptor expression [53,54,55], that could account for specific binding of **[^18^F]2a** and **[^18^F]2b** in the present study. The choroid plexus localize to the inner surface of each lateral ventricle, to the roofs of the third and fourth ventricles, and to the parts of the fourth ventricle that are located caudally and ventrally to the cerebellum. They originate from the ependymal layer and are highly vascularized. Their main role is the production of the cerebrospinal fluid (CSF). The gateway into the CSF are the choroid plexus themselves. They form the blood-CSF barrier that consists of fenestrated endothelial cells and is much less selective than the blood-brain-barrier [56,57].

In contrast to the previously reported specific binding of a radioligand in the pituitary gland [35], binding in the ventricular system has as yet not been imaged by PET in vivo with radioligands of the carboxamide type. Accumulation of carboxamides in the ventricular CSF has, however, been reported in ex vivo autoradiography studies [35,58,59]. Hocke et al. suggested that this phenomenon is due to rapid and active transport by transporters into the CSF, recognizing the diaryl function and non-specific binding at ependymal and plexus tissue [59]. Höfling et al. put forth the hypothesis that the observed binding was indeed at the ependymal layer and D3 specific, since uptake was lowered by 50% in blocking studies with BP897. As unlikely alternative explanation CSF accumulation of a possible radioactive metabolite was also mentioned [58]. Binding to CSF proteins was excluded in in vitro experiments and an unbound state or again binding to ependyma taken into account. Diffusion of tracers into CSF post-mortem was, however, seen as another plausible explanation for tracer accumulation [35]. The present PET study clearly showed specific binding in the ventricular system, disproving the latter hypothesis. The high percentage of free tracer in CSF was also verified in the present evaluation (Figure 3b). Ex Vivo autoradiography did not show homogeneous binding in the ventricles throughout all the sections. There was elevated and displaceable binding in distinct areas of the ventricles consistent with the PET images of **[^18^F]2b**, likely to choroid plexus tissue.

On the one hand, we also tested if **[^18^F]2b** is a substrate for the P-gp transporter to exclude the low initial uptake in cerebral matter through rapid efflux. In contrast to the radiopharmaceutical ^99m^Tc-MIBI (^99m^Tc-methylisobutylnitrile), **[^18^F]2b** was identified as being no P-gp substrate. On the other hand, the rapid transport of extracellular free tracer from the blood to the CSF by penetration of the blood-cerebrospinal fluid barrier, is probably responsible for the lack of visualization of the brain regions rich in D3 receptors by the three radioligands studied.

In the past, no successful PET study selectively visualizing the D3 receptor has been reported in rodents. A study conducted to characterize the in vivo pharmacokinetics and pharmacological properties of [^11^C]PHNO at the D3 receptor in rats used an intracerebral beta-sensitive system [45]. Shortly thereafter, PET studies using [^11^C]PHNO were reported in cats and humans [60,61]. The present study suggests that it is a typical phenomenon in rats that D3 receptor ligands belonging to the carboxamide type are taken up into the ventricular system, simultaneously showing high specific binding in the pituitary gland.

In conclusion, the highly affine and D3 selective compounds **[^18^F]1**, **[^18^F]2a**, and **[^18^F]2b** were synthesized at yields, radiochemical purity, and specific activity acceptable for visualization of dopamine D3 receptors by PET. **[^18^F]2b** can be used for selectively quantifying the concentration of D3 receptors by in vitro autoradiography, due to its markedly lower non-specific binding compared to **[^18^F]1** and **[^18^F]2a**. While the carboxamide **1** revealed low signal/background ratios in the brain and relatively low uptake in the pituitary gland, the azocarboxamides **[^18^F]2a** and **[^18^F]2b** showed interesting characteristics for selective in vivo PET imaging of the dopamine D3 receptor in the pituitary gland in living rats. The high uptake observed in the ventricles was blockable by the D3 receptor ligand BP897, allowing the conclusion that an, however, as yet, uncharacterized specific binding or transport mechanism is involved in ventricular uptake of the radioligands studied herein.

## 4. Material and Methods

### 4.1. General Information

If not otherwise noted, solvents for organic syntheses were of p.a. grade and purchased from Carl Roth GmbH (Karlsruhe, Germany). Chemicals were purchased from Carl Roth GmbH, Sigma Aldrich (Taufkirchen, Germany), Merck (Darmstadt, Germany) or ABCR (Karlsruhe, Germany) in the highest available quality and used without further purification. Special emphasis was on the quality of chemicals and the use of anhydrous solvents for radiosyntheses.

Hydrochlorides were deprotected with saturated solutions of NaHCO_3_ or NaOH. TLC plates (silica gel 60, F_254_, Merck) were bought ready for use and for column chromatography silica gel 60 with a particle size of 40–60 µm was used. The HPLC-System (Series 1100, Agilent, Waldbronn, Germany) was equipped with a VWD UV-Lamp and for radiochemistry was additionally connected to a radio-detector (500 TR Series, Packard-PerkinElmer, Waltham, MA, USA).

Fluorine-18 was provided by PET-Net GmbH (Erlangen, Germany) and delivered as no carrier added (n.c.a.) [^18^F]fluoride, fixed on an anion exchange cartridge (QMA, Waters, Eschborn, Germany) with an average activity range of 0.5–1.0 GBq.

The cultivation of cells was done in a laminar flow hood and based on supplier information. All rat experiments were performed in accordance with protocols approved by the local animal protection authorities (Government of Central Franconia, Ansbach, Germany, No. 54-2532.2-18/12). Rats were housed in groups of two in individual cages in a temperature and air-controlled room with free access to food and water on a 12 h light/dark cycle.

### 4.2. Radiosyntheses

**Figure A4:**
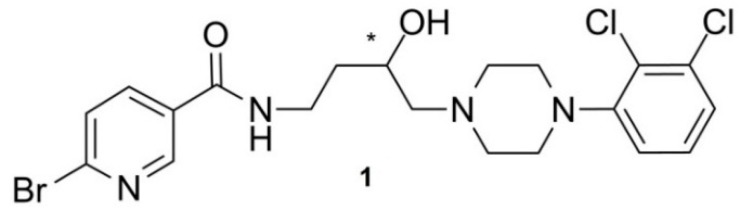
Structure of the precursor **1** needed for the direct labelling strategy of **[^18^F]1**.

The radiosyntheses of **[^18^F]2a** and **[^18^F]2a** has been previously reported [36,38]. The specific activity was determined with HPLC converting the area under curve of the UV-signal, overlapping with the signal of the radiochannel into a molar amount. Therefore a factor was previously determined with a calibration curve ploting a molar amount range of reference compound and corresponding area under the curve. After the radiosynthesis an aliquot of the radioligand reformulated in saline was injected onto the HPLC system and with the factor the detected area under curve could be assigned to a molar amount. The lowermost amount of reference compound used to create the calibration curve was also the detection limit. The synthesis of the precursor **1** (Figure A4) needed for the direct labeling of **[^18^F]1** followed previously published procedures [37,39,62]. ^1^H-NMR (600 MHz, CDCl_3_): δ (ppm) = 1.57–1.67 (m, 1H), 1.77–1.87 (m, 1H), 2.37–2.53 (m, 2H), 2.58–2.68 (m, 2H), 2.86–2.93 (m, 2H), 3.09–3.20 (m, 4H), 3.40–3.48 (m, 1H), 3.85–3.99 (m, 2H), 6.96 (dd, *J* = 7.5, 2.1 Hz, 1H), 7.13–7.19 (m, 2H), 7.40 (dd, *J* = 8.3, 0.7 Hz, 1H), 7.61 (bs, 1H), 8.10 (dd, *J* = 8.3, 2.5 Hz, 1H), 8.78 (d, *J* = 2.4 Hz, 1H). ^13^C-NMR (91 MHz, CDCl_3_): δ (ppm) = 32.80 (CH), 38.96 (CH), 51.37 (2 × CH_2_), 53.23 (2 × CH_2_), 63.58 (CH_2_), 66.97 (COH), 118.62 (CH), 124.21 (CH), 124.86 (CH), 127.49 (CH), 127.61 (Cq.), 129.30 (Cq.), 134.16 (Cq.), 137.80 (CH), 148.18 (CH), 150.95 (Cq.), 154.00 (CO), 164.12 (CBr). HPLC: column: Chromolith RP-18e, 100 mm × 4.6 mm, flow rate: 4 mL/min, solvent A: water (0.1% TFA), solvent B: acetonitrile (0.1% TFA), gradient A/B: 90:10 to 50:50 in 5 min., λ = 214 nm t_R_ = 3.3 min, purity: 99% column: Luna C18(2) 5 µm, 150 mm × 4.6 mm, flow rate: 1.5 mL/min, solvent: A: water (0.1% TFA), solvent B: acetonitrile (0.1% TFA), gradient A/B: 85:15 to 10:90 in 30 min., λ = 254 nm, t_R_ = 9.7 min, purity: 99%.

*N-(4-(4-(2,3-Dichlorophenyl)piperazine-1-yl)-3-hydroxybutyl)-6-fluoronicotinamide* **[^18^F]1**. The synthesis of **[^18^F]1** follows a previously published procedure which was modified [39]. [^18^F]Fluoride was eluted from the QMA-cartrigde with a solution of Kryptofix 2.2.2 (15 mg, 39.8 µmol) and K_2_CO_3_ (1.0 M, 15 µL) in CH_3_CN (900 µL). Water was removed azeotropic by evaporation of CH_3_CN (3 × 500 µL) under a stream of nitrogen at 85 °C. The reactor was removed from the oil bath and the temperature set to 150 °C. When the temperature reached 120 °C **1** (5 mg, 10 µmol) solved in DMSO (400 µL) was added to the dried [K_2.2.2_K^+^]^18^F^−^ complex, and the solution was stirred for 20 min, whilst reaching the end temperature. The reaction solution was diluted with 600 µL (CH_3_CN/H_2_O (0.1% TFA), 1:5) purified by HPLC (Luna (C18), 250 mm × 8 mm, 4 mL/min, solvent: A: H_2_O (0.1% TFA), solvent B: CH_3_CN (0.1% TFA), isocrat. A/B: 67:33, λ = 254 nm, t_R_: 12.4 min) and subsequent SPE (SepPak C18 light, Waters). The product was eluted from the cartridge with ethanol (2 mL), the solvent removed under reduced pressure and the residue dissolved in isotonic saline for further studies.

The identity of **[^18^F]1** was determined using the previously synthesized non-radioactive reference compound [37]. The reference compounds was coinjected with the radioligand for HPLC analysis and the identity confirmed by identical retention times using two different HPLC methods (Method 1: Chromolith RP-18e, 100 mm × 4.6 mm, 4 mL/min, solvent A: water (0.1% TFA), solvent B: acetonitrile (0.1% TFA), gradient A/B: 90:10 to 50:50 in 5 min., λ = 214 nm; Method 2: Luna C18 5 µm, 150 × 4.6 mm, 1.5 mL/min, solvent: A: water (0.1% TFA), solvent B: acetonitrile (0.1% TFA), gradient A/B: 85:15 to 10:90 in 30 min., λ = 254 nm).

### 4.3. Determination of LogD_7.4_, Stability and Binding to Proteins

Human serum was purchased (Biochrom, Biochrom, UK). Human plasma and cerebrospinal fluid was a kind gift from the laboratory of Prof. P. Lewczuk of the clinical neurochemistry in Erlangen. Rat plasma was collected from male Sprague Dawley blood samples. The blood samples were collected intravenous and centrifuged at 2000 rpm for 15 min in heparin coated vials. The supernatant was stored in aliquots at −20 °C. Radioactivity was measured with radio HPLC or in a gamma-counter (Wallac Wizard, PerkinElmer, Waltham, MA, USA). The determination of the distribution coefficient at pH 7.4 (log D_7.4_) has been previously published [38].

#### 4.3.1. Determination of Radioligand Stability

For the in vitro stability, an aliquot of the respective radioligand (1–5 MBq) was added to human serum (200 μL) and incubated at 37 °C. Aliquots (20 μL) were taken after 15, 30 and 120 min and analyzed by radio-HPLC (Method 1).

For the determination of radioligand stability in vivo and detection of radiometabolites the respective radiotracer (15 MBq) was administered intravenous to male Sprague Dawley rats (200–250 g) under isoflurane anesthesia. Blood samples were drawn intravenous after 5, 15 and 30 min and centrifuged for 5 min at 2000 rpm in heparin-coated vials. An aliquot of 50 µL was taken from the supernatant and added to an equal volume of water (10% TFA). The protein precipitate was centrifuged off for 5 min at 25,000 rpm and an aliquot of the supernatant was fractioned with HPLC (Chromolith RP-18e, 100 mm × 4.6 mm, 4 mL/min, solvent A: water (0.1% TFA), solvent B: acetonitrile (0.1% TFA), gradient A/B: 90:10 to 0:100 in 15 min, λ = 214 nm). The fractions were then analyzed in the gamma-counter. To identify the fractions containing the intact radioligand, the retention time and fractioning of the respective tracer was previously assessed. Two independent experiments were performed.

#### 4.3.2. Determination of Binding to Proteins

The free fraction in vitro was determined by adding the respective radioligand (100 kBq) to plasma or CSF (200 µL) and mixed vigorously. Aliquots of 20–50 µL were transferred to size exclusion chromatography columns (MicroSpin G-50; GE Healthcare GmbH, Solingen, Germany, centrifuged for two min at 2000 rpm, and the radioactivity in the filtrate and in the resin were measured in the gamma-counter. Three independent experiments were performed in triplicates.

For the ex vivo determination of the radioligands binding to proteins the respective tracer (15 MBq) was administered intravenous to male Sprague Dawley rats (200–250 g) under isoflurane anesthesia. Blood samples were drawn intravenous after 5, 15 and 30 min and centrifuged for 5 min at 2000 rpm in heparin-coated vials. An aliquot of 50 µL was taken from the supernatant and added to an equal volume of water (10% TFA). The protein precipitate was centrifuged off for 5 min at 25,000 rpm and analyzed together with a further 50 µL aliquot of the supernatant in the gamma-counter. Two independent experiments were performed.

### 4.4. In Vitro Autoradiography

Male Sprague Dawley rats (200–250 g) were sacrificed by decapitation under deep isoflurane anesthesia and brains were removed and frozen in cooled hexane at −70 °C. Coronal brain sections (20 µm) were cut on a cryostat microtome (HM550, Microm-Thermo Fisher Scientific, Walldorf, Germany) and thaw-mounted on covered glass slides (Histobond^®^, Marienfeld, Lauda Königshofen, Germany). The thaw mounted sections were stored at −20 °C up to 14 days.

The cryostat sections were pre-incubated for 15 min in incubation buffer (50 mM Tris-HCl, 40 mM NaCl, pH 7.4) at room temperature. The sections were then placed in incubation buffer containing 5 MBq of the respective tracer. For displacement studies BP897 (50 nM or 1 µM) was added. The sections were incubated at room temperature for 45 min and afterwards washed in ice cold incubation buffer for 3 × 5 min. Subsequently, they were dipped in water and after drying placed on a phosphor imaging screen (Fujitsu, Berlin, Germany) over night. The screens were analyzed with a DUERR Medical HD-CR 35 Bio (Raytest, Straubenhardt, Germany) and evaluated using the software AIDA image analyzer (Raytest). For anatomical identification of brain regions, the sections were compared with figures in the rat brain atlas [42].

### 4.5. Saturation Binding Assay

For saturation-binding assay the cryostat sections (12 µm) prepared as described in the in vitro autoradiography, of eight male Wistar rats (350–400 g) were pre-incubated in buffer (50 mM Tris-HCl, 40 mM NaCl, pH 7.4) for 15 min at room temperature. The sections were then incubated in buffer containing **[^18^F]2b** in concentrations from 0.35–3.5 nM for 45 min at room temperature. For the determination of non-specific binding sections were incubated in the presence of BP897 (1µM). Afterwards the sections were washed with ice cold incubation buffer for 3 × 5 min. Autoradiographic standards were generated by evaporation of aqueous solutions (5 µL) of each concentration on glass slides. The sections were dipped in water and after drying placed on a phosphor imaging screen (Fujitsu) over night, together with the standards. The screens were analyzed as described above. The optical density values were converted to pmol per gram of tissue (wet weight, based on density of 1 g/mL) relative to the standards, with subtraction of non-specific binding, which was calculated by linear regression as a function of ligand concentration. The saturation binding parameters B_max_ and K_d_ were calculated using the software GraphPad Prism (6.0, GraphPad Software, Inc., La Jolla, CA, USA), assuming a one-site non-linear model. Four independent experiments were performed in duplicates.

### 4.6. Dynamic PET-Recordings

Small-animal PET imaging studies were conducted with male Sprague Dawleys rats (250–300 g) on a Inveon microPET scanner (Siemens Healthcare, Erlangen, Germany) under isoflurane (3%) anesthesia. After intravenous injection of **[^18^F]2b** (10–15 MBq), dynamic emission recordings consisting of 13 frames of duration increasing from 30 s to 10 min were acquired to a total of 60 min. For displacement studies BP897 (1 mg/kg) was administered 30 min prior to radioligand injection. Dynamic emission images were corrected for attenuation and decay, and reconstructed by the iterative maximum a posteriori estimation, using software installed in the Inveon PET for dead time and random correction. The dynamic PET scan was then evaluated using the software Amide. The regions of interest were evaluated voxel based and the mean activity in %ID/g of each frame calculated.

### 4.7. Ex Vivo Autoradiographic Study

Male Sprague Dawley rats (250–300 g) were injected with 20 MBq of **[^18^F]2b** intra-venous under isoflurane (4%) anesthesia. For blocking studies BP897 (1 mg/kg) was administerd 30 min prior to radioligand injection. Rats were sacrificed by decapitation 30 min post radioligand injection and brains were removed and frozen in cooled hexane at −70 °C. Coronal and horizontal brain sections (12 µm) were cut on a cryostat microtome (HM550, Microm) and thaw-mounted on covered glass slides (Histobond^®^). The glass slides were placed on a phosphor screen (Fujitsu) over night. The screens were analyzed with a DUERR Medical HD-CR 35 Bio (Raytest) and evaluated using the software AIDA image analyzer (Raytest). Afterwards the sections were HE stained and ascribed to figures in the rat brain atlas [42]. Binding in the regions of interest were evaluated as ratio of the activity in the region of interest through background activity. Binding in the cerebral matter of the respective sections was defined as background activity.

### 4.8. In Vitro Assessment of P-gp Mediated Efflux

**Figure A5:**
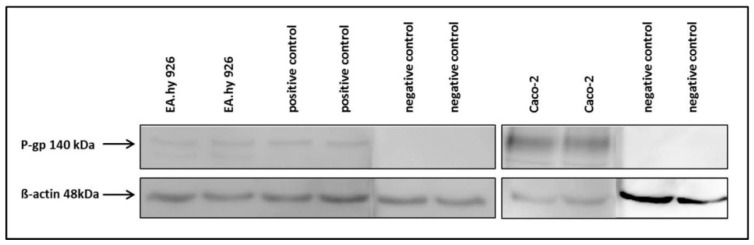
Western blot analysis for expression of P-gp transporter.

**Figure A6:**
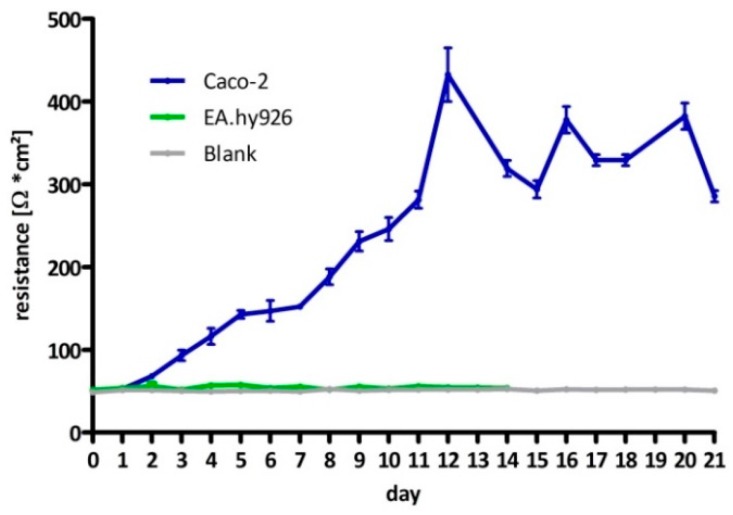
Measurement of trans-epithelial electronic resistance. EA.hy926 were used as negative and blank represents the resistance of the insert membrane without cells.

**Figure A7:**
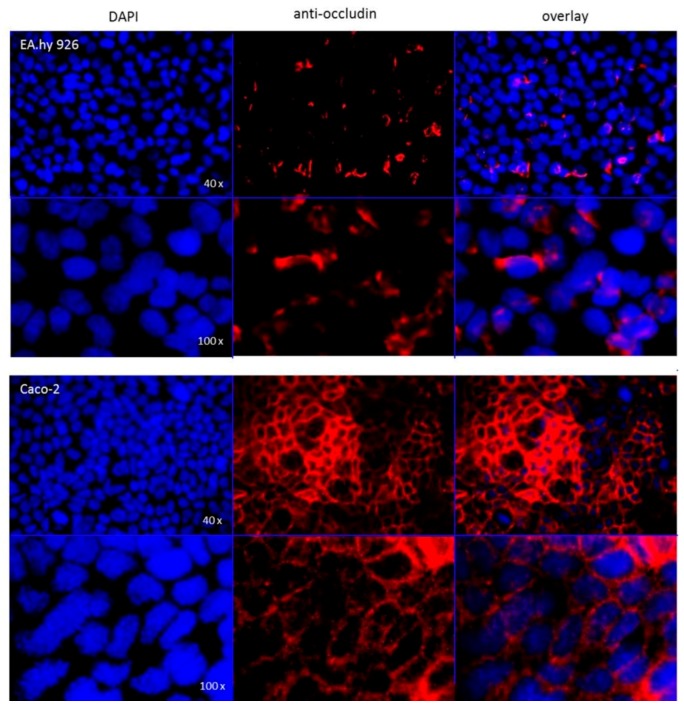
Immunocytochemistry showing formation of tight junctions with Caco-2 cells and Ea.hy926 as negative control. DAPI staining was used to prove formation of monolayer.

The hybrid cell line EA.hy926 was kindly provided by Prof. C. Rödel of the radiation oncology in Erlangen. The Caco-2 cell line was a generous gift from Prof. J. König, Department of Experimental and Clinical Pharmacology and Toxicology at FAU, Erlangen. To guarantee the expression of the P-gp transporter at the current passage a western-blot analysis was performed (Figure A5). For thebi-directional transport assay, formation of tight junctions was followed through measurement of trans-epithelial resistance (Figure A6) and immunocytochemistry targeting occludin (Figure A7).

Commercially available buffers and solutions were bought sterile or sterilized if necessary (Biochrom, Sigma Aldrich). Buffers and medium were kept at 4 °C up to 6 months. All water used was deionized. The cell lines were cultivated at 37 °C in humidified atmosphere containing 5% of CO_2_ under sterile conditions (Heracell, Heraeus-Thermo Fisher Scientific, Hanau, Germany), following supplier information.

Medium EA.hy926: DMEM high glucose, w/o glutamine (FCS 10%, l-glutamine 1%, sodium pyruvate 1%).

Medium Caco-2: EMEM, 2 g/L NaHCO_3_, w/o glutamine (FCS 10%, non-essential amino acids 1%, l-glutamine 1%). The cells were henceforth subcultivated when nearly reaching confluency (3–4 days).

The protein concentration of cell lysates was determined using a BCA-Kit (Sigma-Aldrich, QuantiPro) and a microplate absorbance reader with on board software (iMark, Bio-Rad, München, Germany.

The proteins within the cell lysates (20 µg/lane) were separated by SDS-PAGE. The stacking gel consisted of 5% acrylamide and the separating gel of 7%. The gels and buffers were prepared according to supplier information of the Mini-PROTEAN Tetra Cell System (Bio-Rad).

The primary P-gp antibody (rabbit,1:2500, ab129450, Abcam, Cambridge, UK) was coupled to a secondary HRP conjugated anti-rabbit antibody (goat,1:5000, 401315, Calbiochem) and analysed after short incubation with the detection agent (ECL Prime Western Blotting, GE Healthcare Europe GmbH, Freiburg, Germany) on a Fluor-S Multi imager (Bio-Rad) using the software Quantity One (Bio-Rad). The membrane was washed and primary antibody incubation repeated with β-actin (rabbit, 1:500, A2066, Sigma Aldrich) as loading control. As positive control DU-145 cells were used and the negative control was incubated only with the primary β-actin antibody.

TEER of the Caco-2 cells was determined using the chop-stick method (Millicell-ERS, Merck Millipore, Billerica, MA, USA) following the manufacturers recommendations. All measurements were performed under sterile conditions in a laminar flow hood. Following supplier information Caco-2 cells were seeded near confluency in thin-cert inserts (translucent, Ø 0.4 µm, 24 well PET membrane, Greiner) and placed in multiwell plates (CELLSTAR 24 Multiwell plates, Greiner Bio-One International GmbH, Kremsmünster, Austria). The culture medium basolateral (multiwell) and apical (insert) was renewed every 2–3 days. To determine the day of maximum TEER, on which the experiments were to be carried out, resistance was measured on a daily basis. As negative the EA.hy926 cell line was used and inserts with no cells.

Following application note of the translucent thin-cert inserts (Ø 0.4 µm, 24 well PET membrane, Greiner) an immunocytochemistry targeting occludin was performed after conclusion of the bi-directional transport assay. The occludin antibody (SAB 4200593, Sigma Aldrich) solution (2 µg/mL in 1% FCS/PBS) was applied to each insert and as secondary antibody (IgGCy3, ab6939, Abcam) solution (1 µg/mL in 1% FCS/PBS) was used. For the subsequent nuclear staining, each insert was incubated for 5 min with 100 μL DAPI solution (10 μg/mL). The insert membranes were detached from the insert housings using a scalpel and mounted onto microscopy slides using fluorescence mounting medium. Images were recorded with a fluorescent microscope (DM 6000 B, Leica, Wetzlar, Germany).

#### 4.8.1. Inhibition Assay

EA.hy926 cells were seeded in culture medium at confluency the day prior to experiment or in case of the Caco-2 cells two days prior to experiment in 24-multiwell plates (Greiner Bio-One International GmbH, Kremsmünster, Austria). On the day of the experiment the culture medium was removed and the cells were washed once with PBS (500 µL). Culture medium supplemented with cyclosporine-A (5 µM for EA.hy926 and 10 µM for Caco-2) was added and the cells were incubated at 37 °C for 30 min. Afterwards 1 MBq of **[^18^F]2b** was added to each well and incubated for further 30, 60 and 90 min. After incubation cells were washed with ice cold PBS (500 µL) and lysated with 0.1 M NaOH. The medium and lysates were analysed in the gamma-counter. To identify cyclosporine-A induced accumulation the experiment was also performed using culture medium lacking cyclosporine-A. As positive control the experiment was performed with ^99m^Tc-MIBI (100 kBq/well), a known P-gp substrate [63,64]. To determine the lowermost concentration with which a maximum effect was obtained, cyclosporine-A was applied in a range of 0–50 µM and observed in a time frame of 30–120 min for both cell lines.

#### 4.8.2. Bi-Directional Transport Assay

The procedure of initially adding equal concentrations to both compartments was adapted from the literature [65]. Caco-2 cells were seeded near confluency in thin-cert inserts (translucent, Ø 0.4 µm, 24 well PET membrane, Greiner) and placed in multiwell plates (CELLSTAR 24 Multiwell plates, Greiner). The culture medium basolateral (multiwell) and apical (insert) was renewed every 2–3 days. Approximately 12 days after seeding the Caco-2 cells reached their highest trans-epithelial resistance, which was monitored every four days and on day of the experiment. An immunocytochemistry was also performed with an additional insert (translucent) to confirm stable formation of tight-junctions and cell monolayer. Culture medium was then removed and the cells were washed with PBS. Culture medium supplemented with cyclosporin (10 µM) was added to both compartments and the cells were incubated at 37 °C for 30 min. Afterwards 1 MBq **[^18^F]2b** was added to each compartment. An aliquot was taken from each compartment after 0, 30, 60 and 90 min and analyzed in the gamma-counter. To identify cyclosporine-A induced accumulation the experiment was also performed using culture medium lacking cyclosporine-A. As positive control the experiment was performed with ^99m^Tc-MIBI (100 kBq/ compartment).

## Figures and Tables

**Table 1:** Log D_7.4_ values, along with pKa at the piperazine partial structure. Calculations were performed with the software Marvin Sketch 16.1.11 (ChemAxon Kft., Budapest, Hungary) and experimental values represent the mean ± SD of three independent experiments performed in triplicates.

Compound	Log D_7.4_	Log D_7.4calc_	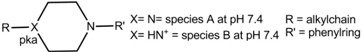
**[^18^F]1**	2.50 ± 0.04	2.63	6.95 (74% A)
**[^18^F]2a**	2.56 ± 0.01	3.89	6.95 (74% A)
**[^18^F]2b**	2.10 ± 0.10	3.17	7.93 (77% B)

**Table 2:** B_max_ of the D3 receptor in rat brain determined with **[^18^F]2b** in four independent experiments. Data are shown as mean ± SD.

Region	B_max_ (pmol/g)	K_d_ (nM)
islands of calleja	20.38 ± 2.67	0.10 ± 0.01
nucleus accumbens	19.54 ± 1.85	0.11 ± 0.07
caudate putamen	16.58 ± 1.63	0.08 ± 0.06

**Figure A3:**
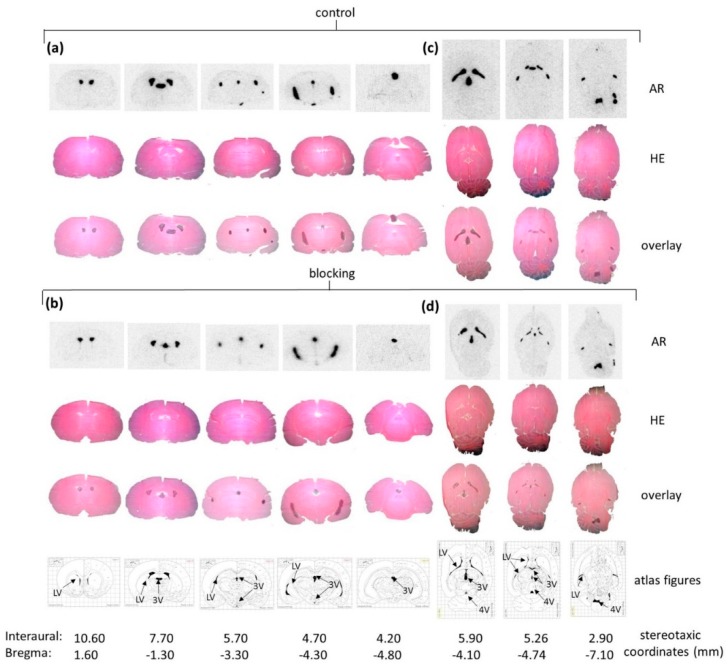
Ex-vivo autoradiography and HE staining results. (**a**) Coronal rat brain sections gathered ex vivo 30 min after administration of **[^18^F]2b**, HE stained and overlayed; (**b**) To (**a**) corresponding coronal rat brain sections with administration of BP897 30 min prior to radioligand administration. HE stained sections were overlayed with the autoradiogram and assigned to Figure in the rat brain atlas [42]; (**c**) Horizontal rat brain sections gathered ex vivo 30 min after administration of **[^18^F]2b**, HE stained and overlayed; (**d**) To (**c**) corresponding horizontal rat brain sections with administration of BP897 30 min prior to radioligand administration. HE stained sections were overlayed with the autoradiogram and assigned to Figure in the rat brain atlas [42]. Autoradiogram (AR), hematoxilin and eosin staining (HE), lateral ventricle (LV), third ventricle (3V) and fourth ventricle (4V).

## Abbreviations

The following abbreviations are used in this manuscript:

CSF	Cerebrospinal fluid
DOAJ	Directory of open access journals
i.v.	Intravenous
ROI	Region of interest
TAC	Time-activity curve
PET	Positron emission tomography
